# Lipopolysaccharide-binding protein and future Parkinson’s disease risk: a European prospective cohort

**DOI:** 10.1186/s12974-023-02846-2

**Published:** 2023-07-21

**Authors:** Yujia Zhao, Douglas I. Walker, Christina M. Lill, Bastiaan R. Bloem, Sirwan K. L. Darweesh, Brismar Pinto-Pacheco, Brooklyn McNeil, Gary W. Miller, Alicia K. Heath, Myrthe Frissen, Dafina Petrova, Maria-Jose Sánchez, María-Dolores Chirlaque, Marcela Guevara, Maurizio Zibetti, Salvatore Panico, Lefkos Middleton, Verena Katzke, Rudolf Kaaks, Elio Riboli, Giovanna Masala, Sabina Sieri, Raul Zamora-Ros, Pilar Amiano, Mazda Jenab, Susan Peters, Roel Vermeulen

**Affiliations:** 1 Institute for Risk Assessment Sciences, Utrecht University, Nieuw Gildestein, Room 3.53, Yalelaan 2, 3584 CM Utrecht, The Netherlands; 2 Gangarosa Department of Environmental Health, Rollins School of Public Health, Emory University, Atlanta, USA; 3 Institute of Epidemiology and Social Medicine, University of Münster, Münster, Germany; 4 Ageing Epidemiology Research Unit (AGE), School of Public Health, Imperial College London, London, UK; 5 Department of Neurology, Donders Institute for Brain, Cognition and Behaviour, Center of Expertise for Parkinson & Movement Disorders, Radboud University Medical Center, Nijmegen, The Netherlands; 6 Department of Environmental Medicine and Public Health, Icahn School of Medicine, Mount Sinai, New York, USA; 7 Department of Environmental Health Sciences, Mailman School of Public Health, Columbia University, New York, USA; 8 Department of Epidemiology and Biostatistics, School of Public Health, Imperial College London, London, UK; 9 Escuela Andaluza de Salud Pública, Granada, Spain; 10 Instituto de Investigación Biosanitaria Ibs.GRANADA, Granada, Spain; 11 Centro de Investigación Biomédica en Red de Epidemiología y Salud Pública (CIBERESP), Madrid, Spain; 12 Department of Epidemiology, Regional Health Council, IMIB-Arrixaca, Murcia University, Murcia, Spain; 13 Instituto de Salud Pública y Laboral de Navarra, Pamplona, Spain; 14 Navarra Institute for Health Research (IdiSNA), Pamplona, Spain; 15 Department of Neuroscience “Rita Levi Montalcini”, University of Turin, Turin, Italy; 16 SC Neurologia 2U, AOU Città Della Salute E Della Scienza, Turin, Italy; 17 Dipartimento di Medicina Clinica e Chirurgia, Federico II University, Naples, Italy; 18 Public Health Directorate, Imperial College NHS Healthcare Trust, London, UK; 19 Division of Cancer Epidemiology C020, German Cancer Research Center (DKFZ), Heidelberg, Germany; 20 Cancer Epidemiology and Prevention Research Unit, School of Public Health, Imperial College London, London, UK; 21 Clinical Epidemiology Unit, Institute for Cancer Research, Prevention and Clinical Network (ISPRO), Florence, Italy; 22 Epidemiology and Prevention Unit, Fondazione IRCCS Istituto Nazionale dei Tumori di Milano, Milan, Italy; 23 Unit of Nutrition and Cancer, Cancer Epidemiology Research Programme, Catalan Institute of Oncology (ICO), Bellvitge Biomedical Research Institute (IDIBELL), L’Hospitalet de Llobregat, Barcelona, Spain; 24 Sub Directorate for Public Health and Addictions of Gipuzkoa, Ministry of Health of the Basque Government, San Sebastián, Spain; 25 Epidemiology of Chronic and Communicable Diseases Group, Biodonostia Health Research Institute, San Sebastián, Spain; 26 Nutrition and Metabolism (NME) Branch, International Agency for Research on Cancer (IARC-WHO), Lyon, France; 27 University Medical Centre Utrecht, Utrecht, The Netherlands; 28 Institute for Risk Assessment Sciences, Utrecht University, Nieuw Gildestein, Room 3.59, Yalelaan 2, 3584 CM Utrecht, The Netherlands

**Keywords:** Parkinson’s disease, Lipopolysaccharide-binding protein, Pre-diagnostic, Systemic inflammation, Endotoxemia

## Abstract

**Introduction:**

Lipopolysaccharide (LPS) is the outer membrane component of Gram-negative bacteria. LPS-binding protein (LBP) is an acute-phase reactant that mediates immune responses triggered by LPS and has been used as a blood marker for LPS. LBP has recently been indicated to be associated with Parkinson’s disease (PD) in small-scale retrospective case–control studies. We aimed to investigate the association between LBP blood levels with PD risk in a nested case–control study within a large European prospective cohort.

**Methods:**

A total of 352 incident PD cases (55% males) were identified and one control per case was selected, matched by age at recruitment, sex and study center. LBP levels in plasma collected at recruitment, which was on average 7.8 years before diagnosis of the cases, were analyzed by enzyme linked immunosorbent assay. Odds ratios (ORs) were estimated for one unit increase of the natural log of LBP levels and PD incidence by conditional logistic regression.

**Results:**

Plasma LBP levels were higher in prospective PD cases compared to controls (median (interquartile range) 26.9 (18.1–41.0) vs. 24.7 (16.6–38.4) µg/ml). The OR for PD incidence per one unit increase of log LBP was elevated (1.46, 95% CI 0.98–2.19). This association was more pronounced among women (OR 2.68, 95% CI 1.40–5.13) and overweight/obese subjects (OR 1.54, 95% CI 1.09–2.18).

**Conclusion:**

The findings suggest that higher plasma LBP levels may be associated with an increased risk of PD and may thus pinpoint to a potential role of endotoxemia in the pathogenesis of PD, particularly in women and overweight/obese individuals.

**Supplementary Information:**

The online version contains supplementary material available at 10.1186/s12974-023-02846-2.

## Introduction

Parkinson’s disease (PD) is the second most common neurodegenerative disease affecting more than 1% of the population aged 60 years and older [1]. The pathogenesis is complex and multifaceted, with increasing evidence suggesting that neuroinflammation likely plays a fundamental role [2]. Mounting evidence suggests a crosstalk between brain inflammation and peripheral inflammation [2]. Lipopolysaccharide (LPS, also known as endotoxin), the component of the outer membrane of Gram-negative bacteria, is a potent activator of innate immune responses. LPS normally presents at much higher concentrations in the human gut as compared to blood [3]. It has been speculated that already during the early stages of PD, LPS increasingly enters the blood due to a disrupted intestinal barrier, possibly induced by altered gut microbiota [4]. The resultant systemic inflammation, induced by elevated circulating LPS, might in turn exacerbate ongoing neurodegeneration in PD by reinforcing microglial activation in the brain [5]. Thus, endotoxemia has been hypothesized as a potential pathological mechanism of PD [6].

Although accumulating animal studies indicate the involvement of the systemic innate immune response in PD [5], the associated components and underlying mechanism in humans remain unclear. The LPS-binding protein (LBP) is a secretory acute-phase protein synthesized mainly in the liver. LBP has the dual role of promoting innate immune responses to LPS [7], as well as enhancing the neutralization and clearance of LPS by high-density lipoprotein [8]. Given technical limitations of measuring LPS in biofluids with sufficient accuracy [9], elevated LBP concentrations in serum or plasma have been suggested as a useful marker indicating endotoxemia and systemic inflammation in chronic diseases [10–12]. Elevated LBP blood concentration was also recently proposed as a biomarker for intestinal permeability [13].

Several studies have reported lower LBP blood levels in prevalent PD cases as compared to healthy controls [14–18] (Additional file 1: Table S1). However, these studies were mostly based on small groups of PD patients from hospitals. Moreover, LBP levels were assessed after the diagnosis, which might be subject to reverse causation. To the best of our knowledge, no prospective study has yet been conducted on the possible relation between LBP and the risk of PD. Therefore, we aimed to investigate the association between plasma LBP levels and the future PD onset measuring LBP levels in pre-diagnostic plasma samples in a large prospective European cohort.

## Methods

### Study design

The European Prospective Investigation into Cancer and Nutrition (EPIC) is a large prospective cohort study that was initiated in 1992 and recruited more than half a million people in ten European countries. At the time of enrollment, information on diet and lifestyle was collected through validated questionnaires, anthropometric measurements were performed, and blood samples were collected [19]. The EPIC study was approved by the ethical committee of the International Agency for Research on Cancer (IARC) and by the ethical review boards of each study center. All participants signed a written informed consent.

The EPIC4PD study was conducted within EPIC and aimed to prospectively assess the role of risk factors in PD. This sub-study was based on a source population of 220,494 subjects from Sweden, the United Kingdom (UK), the Netherlands, Germany, Spain, Italy and Greece [20]. Potential PD cases were identified through medical record linkage and further validated by experts in movement disorders through clinical records, according to the diagnostic criteria of the UK Brain Bank. Differential diagnosis with other movement disorders (multiple system atrophy, progressive supranuclear palsy, vascular parkinsonism, dementia with Lewy bodies, essential tremor, PD with essential tremor, unclassifiable parkinsonism) was conducted by neurologist experts. A total of 881 PD cases were ascertained. Cases who received a diagnosis after the date of recruitment were defined as incident cases (*n* = 734) [20].

Reliability of diagnoses was determined by the quality of clinical data (rated as ‘poor’, ‘good’ or ‘excellent’), as well as the confidence degree of the expert neurologist’s final judgement (rated as ‘low’, ‘medium’ or ‘high’) [20]. Diagnoses were defined as ‘definite’ only when the confidence degree of the neurologist was high and the data quality was excellent; ‘very likely’ when the confidence degree was high, but data quality was either good or poor; ‘probable’ when the confidence degree was medium and data quality was either excellent or good; and diagnoses were defined as ‘possible’ in all remaining cases.

Here, we conducted a nested case–control study within the EPIC4PD cohort. A total of 352 incident PD cases for whom a plasma sample was available were included for current analyses. No subjects from Sweden and Greece were included as no blood samples were available in the central EPIC biobank. Among the included cases, 45 and 144 were categorized as ‘definite’ and ‘very likely’ cases, respectively. One control per case was selected by incidence density sampling matched by age at recruitment, sex and study center.

### Lipopolysaccharide-binding protein measurement

A blood sample was obtained from each participant at recruitment and stored at a central biobank in liquid nitrogen (at − 196 °C). LBP in plasma was measured by enzyme linked immunosorbent assay (ELISA) (HK315-02, Hycult Biotech) in duplicates. The arithmetic mean of the duplicate measurements for each participant was calculated and used in the statistical analyses. Relative standard deviation (RSD) was calculated to check the variability of the two measurements. The ELISA assays were run on 11 plates, with samples from 64 subjects in each. All pairs of PD case and matched control were measured on the same plates to avoid batch effects in the case–control comparison. The median for RSD of duplicate measurements for one subject was 4.7% (interquartile range 1.9–9.6%), and the RSD of average LBP levels among all subjects was 57.9%.

### Statistical analysis

Conditional logistic regression for the matched case–control sets was applied to investigate the association between plasma LBP levels and PD. LBP levels were naturally log-transformed to reduce influence of extreme values. Smoking is a well recognized inverse risk factor for PD [21], and increased body mass index (BMI) is indicated associated with higher PD incidence [22]. Further considering being overweight and a smoker are related to higher LBP levels [23], smoking status and BMI were deemed as potential confounders and adjusted for in our models. Subjects with missing information on smoking status (*n* = 10 for cases, *n* = 13 for controls) were coded as ‘unknown’.

To assess possible effect modification by sex, we performed stratified analyses on men and women. We also stratified analyses by smoking status at recruitment (current, former, never smokers) and BMI (BMI < 25 kg/m^2^, BMI ≥ 25 kg/m^2^), for which mixed effects logistic regression was used, adjusted for the matching variables (age at recruitment, sex, study center) and BMI/smoking status as fixed effects. ELISA plate was treated as random effect since batch effects might exist given different RSDs in analytical plates (Additional file 1: Fig. S1).

To investigate LBP levels in different stages prior to diagnosis, we ran mixed linear regression analyses on the pre-diagnostic periods (defined as time between recruitment and PD diagnosis) and natural log LBP levels among PD cases.

Furthermore, we performed sensitivity analyses: (i) excluding PD cases diagnosed within 8 years (median) after recruitment to rule out possible reverse causality; (ii) including only definite and very likely PD diagnosis; (iii) excluding case–control pairs in the two plates of ELISA where the RSDs for duplicate LBP measurements were relatively high (Additional file 1: Fig. S1); and (iv) we additionally adjusted for six main food categories (fruit, vegetables, meat, cereal, dairy, fish/seafood intake) one-by-one to account for possible impact of diet on microbiota and LBP.

## Results

A total of 352 PD cases (55% males) and 352 matched controls were included in this study (Table 1). The median age at recruitment was 60 years, and the median period between recruitment and PD diagnosis was 7.8 years. Smoking status and BMI at recruitment were not significantly different between PD cases and controls.

**Table 1:** Characteristics of study participants

Characteristic	PD cases *n* = 352	Controls *n* = 352
Age at recruitment, years^a^	60.8 (54.8–65.7)	60.4 (55.0–65.2)
Age at PD diagnosis, years	68.7 (62.8–74.0)	–
Years between recruitment and PD diagnosis	7.8 (4.6–11.0)	–
Sex, *n* (%)^a^		
Male	195 (55%)	195 (55%)
Female	157 (45%)	157 (45%)
Country, *n* (%)^a^		
Italy	54 (15%)	54 (15%)
Spain	97 (28%)	97 (28%)
UK	142 (40%)	142 (40%)
Netherlands	13 (4%)	13 (4%)
Germany	46 (13%)	46 (13%)
Smoking status at recruitment, *n* (%)		
Never smokers	183 (52%)	174 (49%)
Former smokers	116 (33%)	110 (31%)
Current smokers	43 (12%)	55 (16%)
Unknown	10 (3%)	13 (4%)
BMI categories, n (%)		
BMI < 25 kg/m^2^	119 (34%)	137 (39%)
BMI ≥ 25	233 (66%)	215 (61%)

Continuous data were expressed as median (interquartile range)

Categorical data were expressed as number (percentage)

*BMI* body mass index—calculated as the weight in kilograms divided by the square of the height in meters

^a^Matching variables

Plasma LBP levels were right-skewed distributed (Additional file 1: Fig. S2). LBP levels were slightly higher in PD cases than in controls (median (interquartile range), 26.9 (18.1–41.0) vs. 24.7 (16.6–38.4) µg/ml). LBP concentrations in overweight/obese subjects (BMI ≥ 25 kg/m^2^) were significantly higher than in normal-weight subjects (BMI < 25 kg/m^2^) (median (interquartile range), 27.2 (18.1–42.7) vs. 23.3 (16.5–37.1) µg/ml; *p*-value 0.004).

A positive association was observed between LBP levels at baseline and future PD diagnosis (Table 2). The adjusted odds ratio (OR) was 1.46 (95% confidence interval (CI) 0.98–2.19) per one natural log unit increase in LBP levels. Stratified by sex, elevated LBP levels were associated with PD incidence among women (OR 2.68, 95% CI 1.40–5.13), while for men the OR was 0.93 (0.55–1.59) (*p*-value for interaction 0.016). The association among never smokers was slightly stronger (OR 1.30, 95% CI 0.87–1.95) than in current and former smokers but there was no evidence for effect modification (*p*-value for interaction > 0.05). In contrast, BMI significantly modified the association between LBP and PD (*p*-value for interaction 0.018). LBP was positively associated with incident PD in overweight/obese subjects (OR 1.54, 95% CI 1.09–2.18), but not in normal-weight counterparts (OR 0.78, 95% CI 0.48–1.28). Sensitivity analyses did not materially change the observed associations (Table 2). Additional adjusting for food intake revealed similar results compared with the main analysis (data not shown).

**Table 2:** Association between plasma LBP levels and risk of PD

	PD cases plasma LBP (µg/ml)^a^/n	Controls plasma LBP (µg/ml)^a^/n	Odds ratio (95% CI)^b^	*p*-value
Main analysis^c^	26.9 (18.1–41.0)/352	24.7 (16.6–38.4)/352	1.46 (0.98–2.19)	0.061
Subgroup analysis by sex^c^				
Men	24.6 (18.1–38.9)/195	24.2 (16.9–39.3)/195	0.93 (0.55–1.59)	0.797
Women	29.6 (18.2–44.0)/157	25.4 (16.1–37.9)/157	2.68 (1.40–5.13)	0.003
Subgroup analysis by smoking status^d^				
Current smokers	29.7 (19.1–42.6)/43	23.0 (16.3–43.5)/55	1.12 (0.49–2.51)	0.793
Former smokers	24.2 (17.2–38.1)/116	25.4 (16.6–37.7)/110	1.09 (0.65–1.84)	0.736
Never smokers	27.8 (18.2–40.9)/183	24.4 (17.1–37.0)/174	1.30 (0.87–1.95)	0.198
Subgroup analysis by BMI^d^				
BMI < 25 kg/m^2^	22.7 (15.8–33.3)/119	24.5 (16.7–38.0)/137	0.78 (0.48–1.28)	0.331
BMI ≥ 25 kg/m^2^	29.6 (19.4–43.9)/233	24.8 (16.6–38.4)/215	1.54 (1.09–2.18)	0.016
Exclude cases diagnosed within 8 years^e^	23.7 (16.3–40.1)/169	23.9 (16.1–37.3)/169	1.45 (0.79–2.65)	0.234
Include definite and very likely cases only^e^	24.8 (17.1–40.8)/189	24.7 (16.6–39.6)/189	1.19 (0.69–2.07)	0.532
Exclude ELISA plates with high variability between duplicates^c^	26.4 (18.2–39.7)/288	24.2 (16.6–36.8)/288	1.55 (0.99–2.41)	0.053

^a^Median (interquartile range)/the number of subjects

^b^Odds ratio for one unit increase of naturally logarithmic LBP

^c^Conditional logistic regression for the matched case–control sets, adjusted for BMI and smoking status

^d^Mixed effects logistic regression, adjusted for age at recruitment, sex, study center, as well as BMI or smoking status when applicable, ELISA plate as random effect

^e^Mixed effects logistic regression, adjusted for age at recruitment, sex, study center, BMI and smoking status, ELISA plate as random effect

**Fig. 1:**
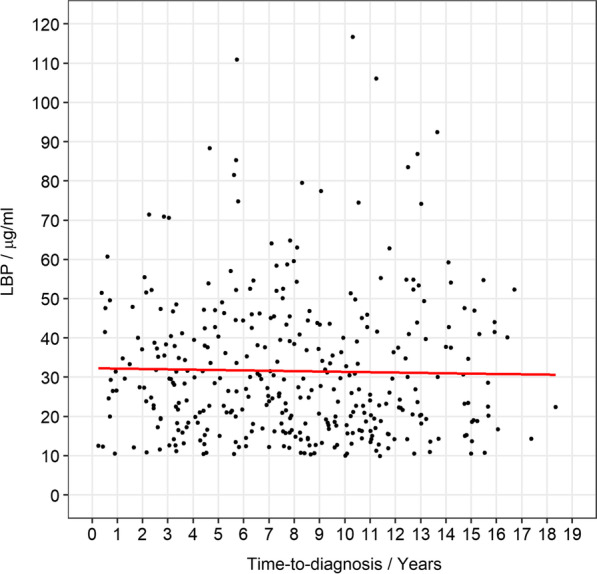
Scatter plot of pre-diagnostic plasma LBP levels in PD cases only (*n* = 352) in relation to years to diagnosis. The line represents the linear regression fitted on log-transformed LBP levels (*β* − 0.004)

There was no obvious trend for plasma LBP levels in different pre-diagnostic periods among PD cases (Fig. 1). The coefficient estimate between logarithmic LBP and year to diagnosis from the linear regression was − 0.004 (95% CI − 0.015 to 0.007).

## Discussion

This is the first study to investigate the association between pre-diagnostic LBP levels and PD risk, utilizing a large European-wide prospective cohort. LBP levels at recruitment were higher in those who subsequently developed PD, which was most pronounced among women and overweight/obese subjects. No differences in LBP levels were observed in different pre-diagnostic periods.

LBP is an acute-phase protein synthesized mainly by hepatocytes. It binds and transfers LPS in blood to the cellular receptor complex consisting of CD14, MD2 and Toll-like receptor 4, resulting in the activation of immune cells and production of inflammatory factors [7]. The levels of LBP rapidly increase when LPS enters the circulation, even at a subclinical level [24]. LBP is more stable than LPS in blood (half-life, 12–24 h [25] vs. 2–4 min [26]) and can be easily measured with immunoassay, making it a good indicator of exposure to LPS.

Elevated LBP levels in blood have been shown to be associated with a higher risk of diverse chronic diseases, including cardiovascular disease [10], allergy [27], arthritis [12], Crohn’s disease [11], and metabolic syndrome [28]. Similar to our findings (median level 26.9 µg/ml), LBP concentrations in patients from these studies were around 20 µg/ml, which are much lower than the levels in acute-phase response (> 100 µg/ml) [29]. These low but increased LBP levels possibly reflect low-grade endotoxemia and chronic systemic inflammation. Chronic systemic inflammation, which, unlike acute inflammation, fails to resolve and leads to a persistent and chronic state [7], has been well acknowledged to be involved in PD pathogenesis [5]. Our findings, in particular those among women and overweight/obese individuals, provide further support for the role of low-grade chronic inflammation in the development of PD.

Excess LPS in the circulation has been hypothesized to result from leaky gut in PD due to intestinal barrier dysfunction. A few preliminary studies observed reduced tight junction protein expression in colonic samples of PD patients [18, 30, 31], and some studies reported increased intestinal permeability in patients [14, 18, 32, 33]. Changes of gut microbiota composition may cause alterations in the gut barrier function and permeability. Recent studies have reported depletion of bacteria in the *Lachnospiraceae* family and the *Faecalibacterium* genus [34], which produce butyrate that is a fundamental energy source for intestinal epithelial cells and plays a role in the maintenance of colonic homeostasis [35, 36]. Bacterial products such as LPS in gut lumen gain access to lamina propria and the bloodstream by gut hyperpermeability. In our study, higher LBP levels in PD cases, which are suggested as an indicator for increased intestinal permeability [13], further supports the involvement of LPS from the intestine in PD pathogenesis.

Aside from the inflammation pathway induced by LPS in the bloodstream, a putative mechanism by which compromised intestinal barrier may influence the brain in PD is the vagal pathway. It has been speculated that alpha-synuclein misfolding and aggregation initially start at the intersection of the gut lumen and the enteric nervous system, which are probably triggered by microbes and their products [37]. Alpha-synuclein may then be propagated to neurons in the central nervous system through the vagus nerve via retrograde transport, causing abnormal alpha-synuclein deposits in the brain, which is a hallmark of PD pathology [37].

In our study, circulating LBP levels were higher in overweight/obese than in normal-weight individuals, which is in line with previous findings from general populations in China [28, 38] and Spain [23]. Effect modification of BMI of the association between LBP and PD risk was noted, and the association was strongest among overweight/obese individuals. This phenomenon is reasonable due to the low-grade chronic inflammation in the development of obesity [39], and gut microbiota is considered as one of the factors in the process. Many experimental studies demonstrated altered microbiota composition, enhanced intestinal permeability, low-grade endotoxemia in animal models of obesity [40–42]. However, the exact biological mechanisms of obesity in the crosslink between gut and PD need further exploration.

Several epidemiological studies have reported an association between LBP levels in serum or plasma and the presence of PD (Additional file 1: Table S1) [14–18]. However, contrary to our results, they all found lower LBP levels in clinically manifest PD compared to controls. Therefore, the difference in LBP is possibly driven by the consequences of the disease (e.g., chronic constipation that is typical of PD, changes in diet, medications) and does not necessarily contribute to the occurrence of the disease (the average disease duration in these studies ranged from 2 to 9.5 years [14–18]). One study indicated that LBP could be transported from the systemic circulation to the intestinal epithelial basolateral and finally into the gut lumen during endotoxemia [43]. This transintestinal efflux possibly explains lower LBP levels in prevalent PD cases, especially when the disease progresses, and the intestinal barrier function deteriorates further. The mechanisms underlying discordant blood LBP levels before and after PD onset remain to be elucidated.

A limitation of our study is that we did not simultaneously analyze proinflammatory cytokines, systemic endotoxin, and intestinal barrier function, thus we could not verify the mechanistic associations between intestinal microbiota composition, increased intestinal permeability, and LPS invasion. Second, our study was performed in patients whose samples were taken during the prodromal phase of PD, on average eight years prior to the diagnosis. Constipation occurs as early as 20 years before the onset of motor symptoms [44], and we have no information about the presence of bowel dysfunction in our participants at the time of sample collection. Therefore, we cannot fully exclude that the higher LBP levels in our study were secondary to PD-related constipation. However, the fact that we see no association between time-to-diagnosis and LBP levels while symptoms worsen closer to diagnosis might speak against this. Furthermore, the observed sex-related discrepancy of LBP levels and PD risk, as the association was significant in women only, suggests the potential presence of distinct sex-dependent pathological mechanisms, which need further confirmation in an independent cohort and warrant further exploration.

## Conclusion

Overall, our results showed that elevated LBP levels prior to diagnosis were associated with higher PD risk in women and overweight/obese individuals. This is the first study to evaluate pre-diagnostic blood LBP levels, shedding some light on LPS-mediated inflammation in the gut–brain axis hypothesis of PD. Future prospective studies are needed to elucidate the association between markers of endotoxemia and the risk of PD.

### Disclaimer

Mazda Jenab—where authors are identified as personnel of the International Agency for Research on Cancer/World Health Organization, the authors alone are responsible for the views expressed in this article and they do not necessarily represent the decisions, policy, or views of the International Agency for Research on Cancer/World Health Organization.

## Supplementary Information

**Additional file 1:** **Table S1.** LBP levels in Parkinson’s disease cases and controls in previous studies. **Figure S1.** Boxplot of relative standard deviations of two LBP measurements from the same subjects, grouped on 11 ELISA plates. **Figure S2.** Histogram with density plot of LBP concentrations on original scale (A) or on natural log scale (B), faceting on controls and Parkinson’s disease cases.

## Data Availability

The datasets used and analyzed during the current study are not publicly available due to privacy agreements.
