# Supplementary material for: Lipopolysaccharide-binding protein and future Parkinson’s disease risk: a European prospective cohort
Source: J Neuroinflammation. 2023 Jul 21;20:170. doi: 10.1186/s12974-023-02846-2 (PMC10362572; doi:10.1186/s12974-023-02846-2)
Supplement: Supplementary file 1 — Additional file 1: Table S1. LBP levels in Parkinson’s disease cases and controls in previous studies. Figure S1. Boxplot of relative standard deviations of two LBP measurements from the same subjects, grouped on 11 ELISA plates. Figure S2. Histogram with density plot of LBP concentrations on original scale (A) or on natural log scale (B), faceting on controls and Parkinson’s disease cases. [file 12974_2023_2846_MOESM1_ESM.docx]

# Additional files

## Table S1. LBP levels in Parkinson’s disease cases and controls in previous studies

| Study | Study design | No. of PD cases | No. of controls | LBP in PD^a^ | LBP in controls^a^ | Test and *p*-value  PD cases vs. controls |
| --- | --- | --- | --- | --- | --- | --- |
| Forsyth 2011 (1) | Case-control | 9 | 10 | 22.9±5.5 | 84.3±31.4 | Mann-Whitney U test  *p*=0.016 |
| Hasegawa 2015 (2) | Case-control | 51 | 36 | 7.8±2.4 | 10.1±5.1 | Student’s t-test  *p*<0.01 |
| Pal 2015 (3) | Case-control | 94 | 99 | 9.3±6.7 | 11.3±7.4 | Analysis of covariance, adjusting age and gender  *p*=0.03 |
| Perez-Pardo 2019 (4) | Case-control | 5 | 5 | 15.73±3.75 | 33.47±6.20 | Student’s t-test  *p*=0.04 |
| Chen 2021 (5) | Case-control | 248 | 149 | 9.08±2.91 | 10.10±3.00 | Mann-Whitney U test  *p*=0.003 |

^a^ Mean ± standard deviation, µg/ml


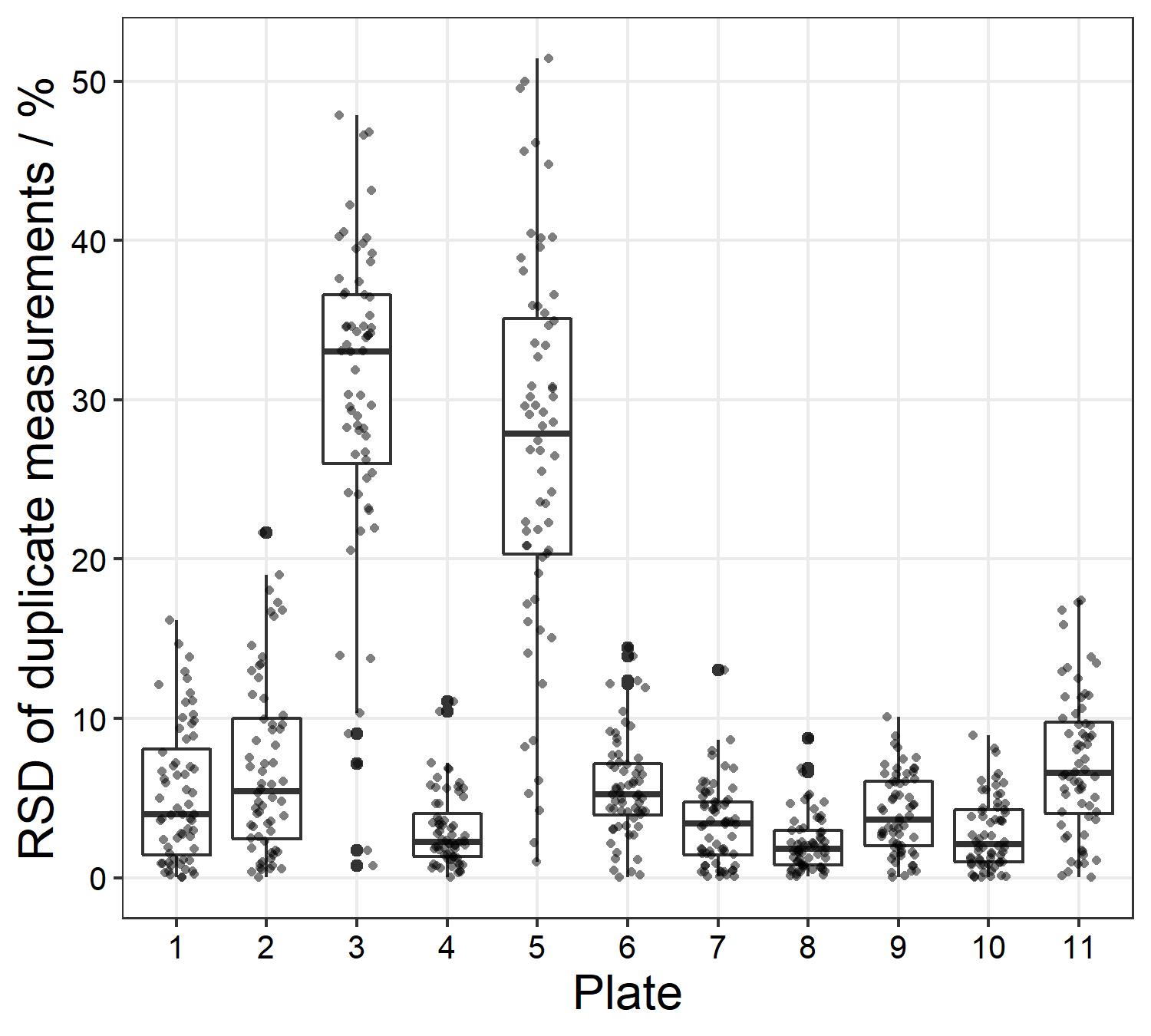


## Figure S1. Boxplot of relative standard deviations of two LBP measurements from the same subjects, grouped on eleven ELISA plates.


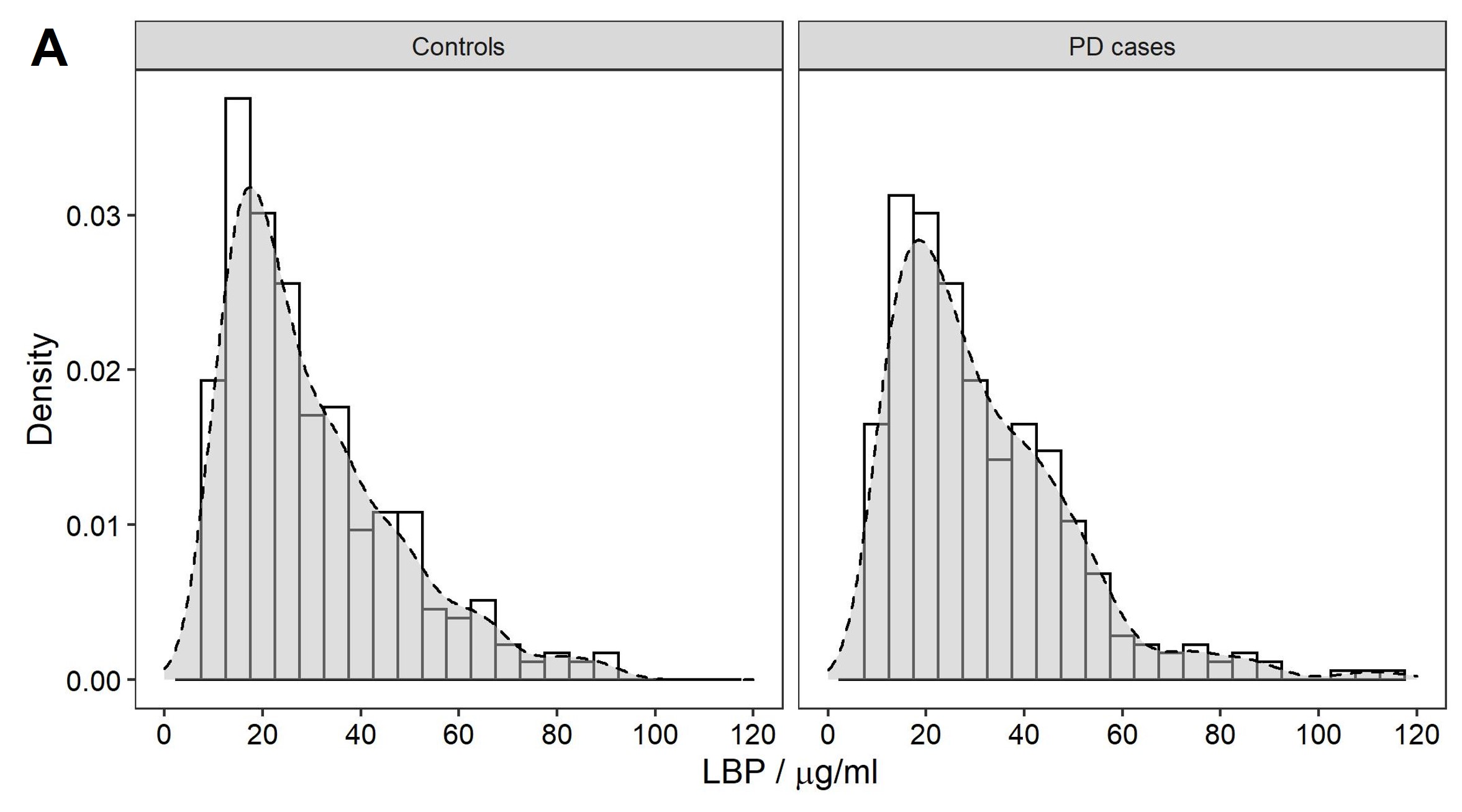


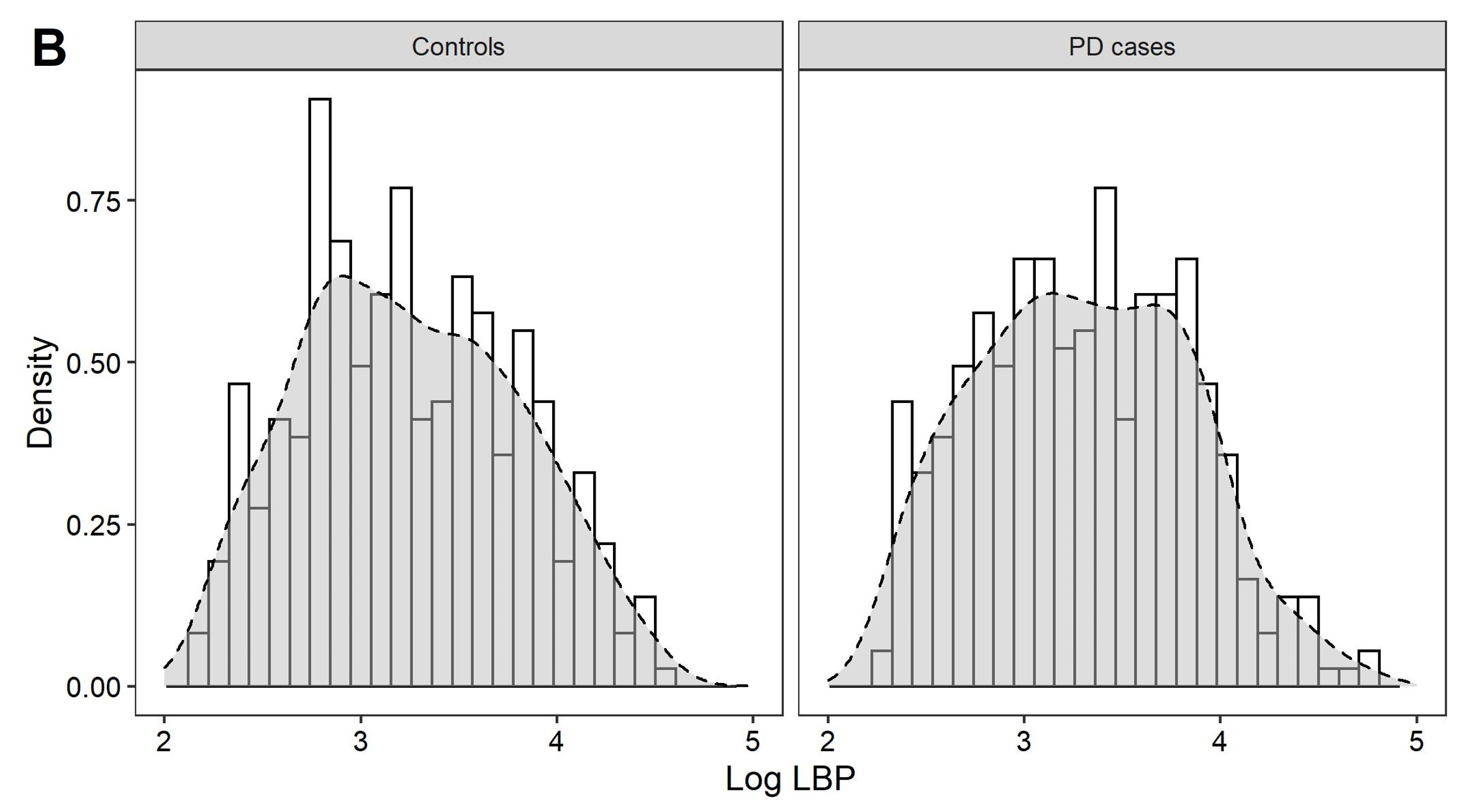


## Figure S2. Histogram with density plot of LBP concentrations on original scale (A) or on natural log scale (B), faceting on controls and Parkinson’s disease cases.

## References

1. Forsyth CB, Shannon KM, Kordower JH, Voigt RM, Shaikh M, Jaglin JA, et al. Increased intestinal permeability correlates with sigmoid mucosa alpha-synuclein staining and endotoxin exposure markers in early Parkinson's disease. PloS one. 2011;6(12):e28032.

2. Hasegawa S, Goto S, Tsuji H, Okuno T, Asahara T, Nomoto K, et al. Intestinal Dysbiosis and Lowered Serum Lipopolysaccharide-Binding Protein in Parkinson's Disease. PloS one. 2015;10(11):e0142164.

3. Pal GD, Shaikh M, Forsyth CB, Ouyang B, Keshavarzian A, Shannon KM. Abnormal lipopolysaccharide binding protein as marker of gastrointestinal inflammation in Parkinson disease. Frontiers in neuroscience. 2015;9:306.

4. Perez-Pardo P, Dodiya HB, Engen PA, Forsyth CB, Huschens AM, Shaikh M, et al. Role of TLR4 in the gut-brain axis in Parkinson's disease: a translational study from men to mice. Gut. 2019;68(5):829-43.

5. Chen SJ, Chi YC, Ho CH, Yang WS, Lin CH. Plasma Lipopolysaccharide-Binding Protein Reflects Risk and Progression of Parkinson's Disease. Journal of Parkinson's disease. 2021;11(3):1129-39.
